# Chemosensitization of multidrug resistant *Candida albicans* by the oxathiolone fused chalcone derivatives

**DOI:** 10.3389/fmicb.2015.00783

**Published:** 2015-08-05

**Authors:** Izabela Ła̧cka, Marek T. Konieczny, Anita Bułakowska, Marie Kodedová, Dana Gašková, Indresh K. Maurya, Rajendra Prasad, Sławomir Milewski

**Affiliations:** ^1^Department of Pharmaceutical Technology and Biochemistry, Gdańsk University of TechnologyGdańsk, Poland; ^2^Department of Organic Chemistry, Medical University of GdańskGdańsk, Poland; ^3^Faculty of Mathematics and Physics, Charles University in PraguePrague, Czech Republic; ^4^Membrane Biology Laboratory, School of Life Sciences, Jawaharlal Nehru UniversityNew Delhi, India

**Keywords:** multidrug resistance, chalcones, antifungals, chemosensitization, *Candida albicans*

## Abstract

Three structurally related oxathiolone fused chalcone derivatives appeared effective chemosensitizers, able to restore in part sensitivity to fluconazole of multidrug-resistant *C. albicans* strains. Compound **21** effectively chemosensitized cells resistant due to the overexpression of the *MDR1* gene, compound **6** reduced resistance of cells overexpressing the ABC-type drug transporters *CDR1/CDR2* and derivative **18** partially reversed fluconazole resistance mediated by both types of yeast drug efflux pumps. The observed effect of sensitization of resistant strains of *Candida albicans* to fluconazole activity in the presence of active compounds most likely resulted from inhibition of the pump-mediated efflux, as was revealed by the results of studies involving the fluorescent probes, Nile Red, Rhodamine 6G and diS-C_3_(3).

## Introduction

Opportunistic fungal infections in immunocompromised hosts have become an important clinical problem, with *Candida* species remaining one of the leading causes of hospital-acquired bloodstream infections. The attributable frequency of deaths from candidemia remains close to 40% and *Candida albicans* comprises nearly half of the isolated fungal pathogens (Pfaller and Diekema, 2007). The main factors determining high mortality from candidal infections are: a limited repertoire of clinically used antimycotics and an emerging appearance of drug resistance, including its multidrug form (Sanglard and Odds, 2002; Pfaller, 2012; Srinivasan et al., 2012). Among molecular mechanisms underlying multidrug resistance (MDR), the most important is an overproduction of membrane proteins belonging to the ATP-binding cassette (ABC) transporters or the major facilitator superfamily (MFS). A number of efflux pumps have been identified in fungi, including Cdr1p, Cdr2p, Mdr1p, and Flu1p in *C. albicans* (Prasad et al., 2002; Prasad and Goffeau, 2012). In view of these facts, the search for new antimycotics active against MDR fungi and/or chemosensitizers, i.e., compounds able to render MDR strains sensitive to clinically used antifungals, is an urgent need. Chemosensitization has been postulated as one of the ways of overcoming fungal resistance to the most popular triazole antifungals, including fluconazole (FLC). Reported examples of compounds effectively chemosensitizing FLC-resistant human pathogenic fungi include Cdr1p/Cdr2p–specific curcumin (Sharma et al., 2009), ibuprofen (Ricardo et al., 2009), or cyclosporine (Marchetti et al., 2000), inhibitors of MFS-type drug transporters, like cerulenin analogs (Diwischek et al., 2009) or synthetic heterocycles containing a cyclobutene-dione core (Keniya et al., 2015) and clorgyline, targeting both types of fungal drug efflux pumps (Holmes et al., 2012).

Chalcones, compounds constituting a subclass of flavonoids, exhibit a number of biological effects, including antimicrobial activity (Dimmock et al., 1999; Nowakowska, 2007). Antifungal properties of some chalcones were demonstrated and it was suggested that the observed activity might be related to the inhibition of biosynthesis of cell wall components, β(1 → 3)glucan and chitin (López et al., 2001). It was also shown that some of the chalcone derivatives inhibited drug extrusion by the yeast drug transporters of the ABC type (Conseil et al., 2000; Wink et al., 2012).

We reported previously that a synthetic oxathiolone fused chalcone derivative AMG-148 exhibited *in* antifungal activity (Ła̧cka et al., 2011). In the present communication, results of our studies on structural analogs of AMG-148, concerning especially their chemosensitizing effect on MDR yeast cells, are described.

## Materials and methods

### Compounds and reagents

The oxathiolone fused chalcone derivatives were synthesized as described (Konieczny et al., 2007a,b,c). Fluconazole was kindly provided by Pliva Krakow (Cracow, Poland). All other chemicals were from Sigma-Aldrich, St. Louis, MO.

### Strains and culture conditions

The reference strain used in this study was *Candida albicans* ATCC 10231. Non-reference strains are listed in Table 1. *C. albicans* F2, F5, B3, B4, Gu4, and Gu5 clinical isolates (Franz et al., 1998, 1999) were kindly provided by J. Morschhäuser, Würzburg, Germany, while DSY2039 and DSY750 by D. Sanglard, Lausanne, Switzerland. *S. cerevisiae* AD1-8u^−^ and US50-18C mutants AD1-3, AD12, AD13, and AD23 were kindly provided by A. Goffeau, Louvain-la-Neuve, Belgium. The AD-derived strains ADCDR1, ADCDR2, and ADMDR1 were constructed by the previously described methods (Gupta et al., 1998; Prasad et al., 1998; Smriti et al., 2002). Strains were grown at 30°C in Sabouraud medium (2% glucose, 1% yeast extract, and 2% bactopeptone) and stored on Sabouraud plates containing 2% agar.

**Table 1:** **Non-reference yeast strains used in this study**.

**Strains**	**Description**	**Source/references**
***SACCHAROMYCES CEREVISIAE***		
US50-18C	*MAT*α*, PDR1-3, ura3, his1* (parent strain)	Balzi et al., 1987
AD1-8u^−^	*MAT*α*, PDR1-3, ura3, his1*, Δ*yor1::hisG*, Δ*snq2::hisG*, Δ*pdr5::hisG*, Δ*pdr10::hisG*, Δ*pdr11::hisG*, Δ*ycf1::hisG*, Δ*pdr3::hisG*, Δ*pdr15::hisG*	Decottignies et al., 1998
ADCDR1	AD1-8u^−^ transformed with *CaCDR1*	Smriti et al., 2002
ADCDR2	AD1-8u^−^ transformed with *CaCDR2*	Smriti et al., 2002
ADMDR1	AD1-8u^−^ transformed with *CaMDR1*	Gupta et al., 1998
AD1-3	*MAT*α*, PDR1-3, ura3, his1*, Δ*yor1::hisG*, Δ*snq2::hisG*, Δ*pdr5::hisG*	Decottignies et al., 1998
AD12	*MAT*α*, PDR1-3, ura3, his1*, Δ*yor1::hisG*, Δ*snq2::hisG*	Decottignies et al., 1998
AD13	*MAT*α*, PDR1-3, ura3, his1*, Δ*yor1::hisG*, Δ*pdr5::hisG*	Decottignies et al., 1998
AD23	*MAT*α*, PDR1-3, ura3, his1*, Δ*snq2::hisG*, Δ*pdr5::hisG*	Decottignies et al., 1998
***CANDIDA ALBICANS* CLINICAL ISOLATES**		
Gu4	Fluconazole sensitive	Franz et al., 1998
Gu5	Fluconazole-resistant due to the overexpression of *CDR1* and *CDR2*	Franz et al., 1998
F2	Fluconazole sensitive	Franz et al., 1999
F5	Fluconazole-resistant due to the overexpression of *CaMDR1* and *ERG11*	Franz et al., 1999
B3	Fluconazole sensitive	Franz et al., 1998
B4	Fluconazole-resistant due to the overexpression of *CaMDR1*	Franz et al., 1998
DSY2039	Fluconazole sensitive	D.S.^a^
DSY750	Fluconazole-resistant due to the overexpression of *CaMDR1*	D.S.

a *strains provided by Dominique Sanglard, Lausanne, Switzerland*.

### Susceptibility testing procedures

MIC values of tested compounds were determined in RPMI-1640 medium by the slightly modified serial dilution microtiter plate method recommended by CLSI (Clinical Laboratory Standards Institute, 2008). Turbidity in individual wells was measured with a microplate reader (Victor^3^V, Perkin Elmer). The MIC was defined as the lowest drug concentration at which at least 80% decrease in turbidity, in comparison to the drug-free control, was observed.

The same conditions were applied for quantification of an antifungal effect of chalcones in combination with Fluconazole (FLC), using the checkerboard microdilution assay. The final concentrations of chalcones ranged from 2 to 64 μg/mL for all chalcones but **11**, for which the concentration range was 0.0625 to 2 μg/mL. FLC was tested in the 0.03125–8 μg/mL range. The data obtained by the checkerboard microdilution assays were analyzed using the model-fractional inhibitory concentration (FIC) index method based on the Loewe theory. The FIC index is defined as the sum of the MIC of each drug when used in combination divided by the MIC of the drug used alone. Synergy and antagonism were defined by FIC indexes of ≤0.5 and >4, respectively. A FIC index value >0.5 but ≤4 was considered indifferent (Odds, 2003).

### ATPase activity assay

The ATPase activity of the plasma membrane fractions was measured in terms of oligomycin-sensitive release of inorganic phosphate, as described previously (Smriti et al., 2002), either alone or in the presence of compounds tested.

### Quantification of energy-dependent rhodamine 6G efflux

Preparation of yeast cells was performed as described previously (Sharma et al., 2009). Rhodamine 6G (R6G) solution was added to 1 ml aliquots of 2% cell suspension in PBS (to get the 10 μM final concentration of R6G) along with the compound tested and the mixtures were incubated for 1 h at 30°C. The cells were washed twice with PBS and re-energized by re-suspending them in 1 ml of PBS containing 2% glucose and incubated at 30°C for 30 min. After incubation, the samples were centrifuged at 9000 × g for 2 min and absorption of the supernatant was measured at 527 nm.

### Nile Red accumulation assay

The accumulation of Nile Red (NR) was determined by modification of the method described elsewhere (Ivnitski-Steele et al., 2009) and measured with a FACSort flow cytometer (Becton-Dickinson Immunocytometry Systems, San Jose, CA). Exponential phase yeast cells were collected, washed 3 × with water and suspended in PBS, pH 7.4, containing 2% glucose to the final cell density 2% (w/v). The NR solution was added to 1 ml portions of the cell suspension in PBS/glucose to get the 7 μM final concentration of NR, along with the compound tested. After 30 min incubation at 30°C, the samples were excited with a 488-nm laser and PE–Texas Red filter was used to detect NR-derived fluorescence. The mean fluorescence intensity was calculated using the histogram stat program. Analysis was performed with the CellQuest software (Becton-Dickinson Immunocytometry Systems).

### DiS-C_3_(3) accumulation assay

Fluorescence measurement of diS-C_3_(3) accumulation in cells was performed using the procedure described previously (Hendrych et al., 2009). Briefly, the fluorescent probe diS-C_3_(3) (final concentration 2 × 10^−8^ M) was added to the cell suspension 10 min after compounds tested and fluorescence emission spectra of the cell suspensions were measured (λ_ex_ = 531 nm) at the time of staining. In each experiment, the CD cocktail (5 μM CCCP plus 10 μM DM-11) was added, usually after 40 min of staining.

### Transmission electron microscopy

*C. albicans* cells from the overnight cultures were harvested, washed and suspended in Sabouraud medium to the final cell density of ≈ 10^6^ cfu/mL. The compounds tested were added and cultures were incubated for 9 h at 30°C. For ultrastructural studies, the cells were fixed with 2% glutaraldehyde in 0.1% phosphate buffer for 3 h at 25°C, washed with 0.1 M phosphate buffer (pH 7.2) and post-fixed with 1% OsO_4_ in 0.1 M phosphate buffer for 1 h at 4°C. Samples were dehydrated with graded acetone, cleared with toluene, infiltered consequently with toluene and araldite mixture at room temperature and pure araldite at 50°C and finally embedded in an Eppendorff tube with pure araldite mixture at 60°C. Semithin and ultrathin section cutting was done with ultramicrotome (Ultramicotome Lecia EM UC6). Sections were taken on the 3.05 mm diameter, 200 mesh copper grid, stained with uranyl acetate.

## Results

### Growth inhibitory effect of chalcone derivatives

In the previous study, AMG-148, an oxathiolone fused chalcone derivative, was found to exhibit *in vitro* antifungal activity against several strains of human pathogenic yeasts, with MIC values within the range of 1–16 μg/mL and a fungicidal effect was observed at concentrations 4–32-fold higher than the MICs (Ła̧cka et al., 2011). In this work, a growth inhibitory effect of AMG-148 (here compound **11**) was compared to that of its 26 structural analogs, using the serial dilution microtiter plate method employing *C. albicans* ATCC 10231 as a reference microorganism. Results presented in Table 2 indicate that all compounds but **11** exhibited poor anticandidal activity, with MICs in the 64 − > 256 μg/mL range. MIC of the known antifungal drug FLC in this assay was 2 μg/mL.

**Table 2:** **Fungistatic activity of oxathiolone-fused chalcones**.

**Compound**	**Structure**						**MIC^a^ (μg mL^−1^) *C. albicans*ATCC 10231**
	**R_1_**	**R_2_**	**R_3_**	**R_4_**	**R_5_**	**R_6_**	
Type 1	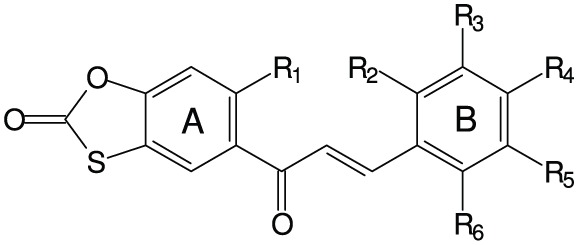						
1	−OCH_3_	−H	−H	−H	−H	−H	64
2	−OCH_3_	−H	−H	−H	−Cl	−H	64
3	−OCH_3_	−H	−H	−OCH_2_CH_2_N(C_2_H_5_)_2_	−H	−H	64
4	−OCH_2_CH_2_N(C_2_H_5_)_2_	−H	−H	−H	−H	−H	128
5	−OCH_2_CH_2_N(C_2_H_5_)_2_	−H	−H	−Br	−H	−H	128
6	−OCH_2_CH_2_N(C_2_H_5_)_2_	−H	−H	−OCH_3_	−H	−H	64
Type 2	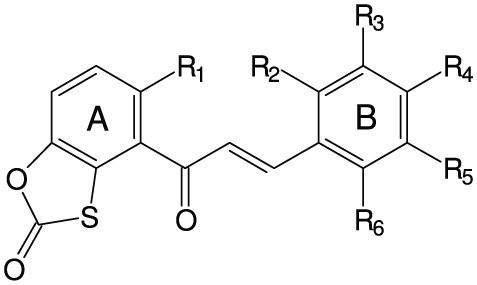						
7	−OCH_3_	−H	−H	−OCH_3_	−OCH_3_	−H	>256
8	−OCH_3_	−H	−H	−N(CH_3_)_2_	−H	−H	>256
9	−OCH_3_	−H	−H	−NO_2_	−H	−H	>256
10	−OCH_3_	−H	−H	−H	−Cl	−H	>256
11	−OCH_3_	−H	−H	−OCH_2_CH_2_N(CH_3_)_2_	−H	−H	2
12	−OCH_2_CH_2_N(C_2_H_5_)_2_	−H	−H	−Cl	−H	−H	128
13	−OCH_2_CH_2_N(C_2_H_5_)_2_	−H	−H	−H	−Cl	−H	128
14	−OCH_2_CH_2_N(C_2_H_5_)_2_	−H	−H	−H	−H	−Cl	128
15	−OCH_2_CH_2_N(C_2_H_5_)_2_	−H	−H	−OCH_3_	−H	−H	64
16	−OCH_2_CH_2_N(C_2_H_5_)_2_	−H	−H	−OCH_2_CH_2_N(CH_3_)_2_	−H	−H	64
17	−OCH_2_CH_2_N(C_2_H_5_)_2_	−H	−H	−H	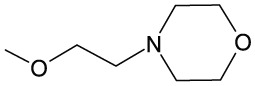	−H	64
18	−OCH_3_	−H	−H	−OCH_2_CH_2_CH_2_N(CH_3_)_2_	−OCH_3_	−H	64
19	−OCH_2_CH_2_CH_3_	−H	−H	−H	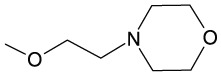	−H	128
20	−OCH_3_	−H	−H	−H	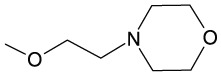	−H	128
21	−OCH_2_CH_2_CH_3_	−H	−H	−OCH_2_CH_2_N(CH_3_)_2_	−H	−H	64
22	−OCH_2_CH_2_N(C_2_H_5_)_2_	−H	−OCH_3_	−OCH_3_	−OCH_3_	−H	128
23	−OCH_2_CH_2_N(C_2_H_5_)_2_	−H	−H	−OCH_3_	−OCH_3_	−H	128
24	−OCH_2_CH_2_CH_3_	−H	−H	−OCH_3_	−H	−H	128
25	−OCH_2_CH_2_CH_3_	−H	−H	−OCH_3_	−OCH_3_	−H	128
26	−OCH_2_CH_2_CH_3_	−OCH_3_	−H	−OCH_3_	−H	−OCH_3_	128
27	−OCH_2_CH_2_N(CH_3_)_2_	−H	−H	−OCH_3_	−H	−H	64

a *MICs were determined in RPMI-1640 buffered medium, as described in Materials and Methods*.

### Combined antifungal effect of chalcone derivatives and fluconazole

Antifungal effect of 11 chalcone derivatives with MIC values ≤ 64 μg/mL (Table 2) in combination with FLC was quantified using the checkerboard serial dilution assay. The only case of a slight synergistic effect was noted for combination of FLC with compound **11**, where a FIC index = 0.22 was determined. For combinations of all the other 10 chalcones tested with FLC, the FIC indexes were in the 0.92–1.36 range, thus indicating neither synergy nor antagonism.

### Modulation of multidrug resistance

Some natural flavonoids and their synthetic derivatives were reported to be effective modulators of microbial multidrug resistance (Ivanova et al., 2008; Liu et al., 2008; Sharma et al., 2010). To check whether chalcones tested in this work were able to restore the antifungal potency of FLC against FLC-resistant human pathogenic yeasts, an *in vitro* assay was performed employing *C. albicans* clinical isolates resistant to fluconazole, due to the FLC-induced overexpression of genes encoding multidrug efflux pumps. The Gu5 and B4 isolates are FLC-resistant, due to the documented overexpression of *CDR1* and/or *CDR2* in the former and *MDR1* in the latter. Their FLC-sensitive counterparts, Gu4 and B3, respectively, exhibit a basal expression of these resistance genes. The antifungal activity of FLC against *Candida* isolates was determined in the presence of a fixed concentration of each chalcone. All compounds were tested at concentrations that did not interfere with fungal growth (< 1/2MIC; 0.5 μg/mL for **11** and 25 μg/mL for the other compounds). Sixteen out of twenty seven chalcones did not show any effect but the remaining 11 were able to decrease the MIC_FLC_ value of at least one of the FLC-resistant isolates (Table 3). Eight derivatives demonstrated ability to enhance sensitivity of *C. albicans* B4 to FLC. This effect was significant in the case of compounds **11**, **18**, and **21**. Seven compounds were able to enhance sensitivity of *C. albicans* Gu5 to FLC, however this change was significant only for compounds **6** and **18**. The chemosensitizing efficiency of compounds **6**, **18**, and **21** is thus comparable to that of the known chemosensitizers of fungal drug efflux pumps, verapamil and trifuoperazine. On the other hand, the observed substantial reduction of MIC_FLC_ of the B4 strain in presence of **11** may result from chemosensitization, but at least in part could be also attributed to the observed synergism between FLC and **11** as antifungals.

**Table 3:** **Influence of chalcones on MIC_FLC_ values determined for *Candida albicans* clinical isolates**.

**Compound**	**MIC of FLC (μg/mL)^a^**			
	**B3**	**B4**	**Gu4**	**Gu5**
−	1	16	4	256
5	1	4	4	256
**6**	1	16	4	**32**
**11**	0.5	**1**	2	128
15	1	8	4	64
**18**	1	**2**	4	**32**
19	1	8	4	256
20	1	16	4	128
**21**	1	**2**	4	64
22	1	16	4	128
23	1	8	4	128
25	1	8	4	128
**VP^b^**	1	**0.5**	4	64
**TFP^b^**	1	**1**	4	128

a *MIC values for FLC were determined by the serial dilution method as described Materials and Methods, in the presence of a fixed concentration of a compound tested (0.5 μg/mL for ***11***and 25 μg/mL for the other compounds)*.

b *Verapamil (50 μg/mL) and trifluoroperazine (20 μg/mL) were used as positive controls*.

*Cases of significant (>4-fold) MIC_FLC_ reduction by a given compound are highlighted in bold*.

Several lower concentrations of compounds listed in Table 3 were examined in order to find the lowest concentrations at which the FLC-sensitizing effect was observed. In the case of *CDR1/CDR2*-overexpressing *C. albicans* Gu5, a two-fold reduction of MIC_FLC_ was found for **6** at 5 μg/mL, while **18** did the same at 6.25 μg/mL. In the case of *C. albicans* B4, compounds **21** and **18** caused the twofold reduction of MIC_FLC_ at 0.25 μg/mL and 0.1 μg/mL, respectively.

Compounds **6**, **18**, and **21** were also tested for their intrinsic antifungal activity against FLC-resistant and FLC-sensitive *C. albicans* clinical isolates. Comparison of MIC or IC_50_ values determined for B3/B4 and Gu4/Gu5 pairs indicates, how the enhanced activity of a particular transporter affects drug susceptibility. Data presented in Table 4 confirm resistance of B4 and Gu5 strains to FLC. On the other hand, the antifungal activity of chalcones was in most cases not affected by presence/absence of drug transporters, except for the slight effect observed for compound **6** in the case of the Gu4/Gu5 pair, while no difference in MIC values was found for **18** and **21**. These results suggest that **18** and **21** are not effluxed by both ABC-type and MFS-type drug transporters of *C. albicans*, while **6** may be a poor substrate of Cdr1p or Cdr2p but not of Mdr1p.

**Table 4:** **Activity of compounds 6, 18, 21 and fluconazole against MDR *C. albicans* clinical isolates and their drug-sensitive counterparts**.

**Strain**	**MIC/IC^a^_50_ (μg/mL)**			
	**6**	**18**	**21**	**FLC**
*C. albicans* B3	64/38.5	64/40.0	64/42.8	1
*C. albicans* B4 (MDR1)	64/37.3	64/41.2	64/43.9	16
*C. albicans* Gu4	32/24.2	64/36.6	64/38.4	4
*C. albicans* Gu5 (CDR1/CDR2)	64/35.5	64/36.2	64/39.6	256

a *MICs and IC_50_s were determined by using RPMI-1640 buffered medium, as described in Materials and Methods*.

### Effect on ATPase activity of Cdr1p/Cdr2p

The effect of selected compounds (**6**, **21**, and **18**) on the ATPase activity of Cdr1p/Cdr2p was studied by determination of the oligomycin-sensitive ATP hydrolysis by plasma membrane preparations isolated from *C. albicans* Gu5 clinical isolate overproducing the ABC pumps. No significant reduction of the ATPase activity in presence of the tested compounds was found up to 50 μg/mL. A very slight reduction, about 20% was noted for **18** (50 μg/mL), however at 25 μg/mL the reduction was lower than 5%. It seems therefore that the oxathiolone-fused chalcones studied are not inhibitors of the ATPase activity of the ABC-type *C. albicans* drug transporters.

### Changes in membrane potential and cell integrity monitored with the diS-C_3_(3) probe

Using a set of five isogenic mutant strains, the effect of selected chalcone derivatives on membrane potential and activity of Pdr5p and Snq2p ABC-type drug exporters in *S. cerevisiae* was tested by the fluorescence method, with diS-C_3_(3) as a probe. Intracellular accumulation of the probe is accompanied by a gradual shift of its λ_max_ toward longer wavelengths (red shift), while any possible efflux results in a blue shift. DiS-C_3_(3) is a substrate for both Pdr5p and Snq2p (Čadek et al., 2004; Hendrych et al., 2009), so that comparison of the probe accumulation curves obtained for Pdr5p- and/or Snq2p-expressing and Pdr5p- and Snq2p-deficient cells measured in the presence of any compound may provide information about its effect on a given drug efflux pump. On the other hand, analysis of the level of staining of pump-deficient cells treated with any compound may reveal its influence on membrane potential, as the blue shift indicates plasma membrane depolarization, while a red shift is usually a consequence of hyperpolarization or permeabilization of the cell membrane. Finally, the cell destruction upon the action of any compound may be confirmed by the consequences of inclusion of the CD cocktail (5 μM CCCP with 10 μM DM-11) into the diS-C_3_(3) assay. Addition of the lipophilic, weak acid (CCCP) plus the H^+^-ATPase blocker (DM-11) results in the rapid blue shift for the suspension of intact cells, while the shift does not occur if the cells are broken.

**Figure 1:**
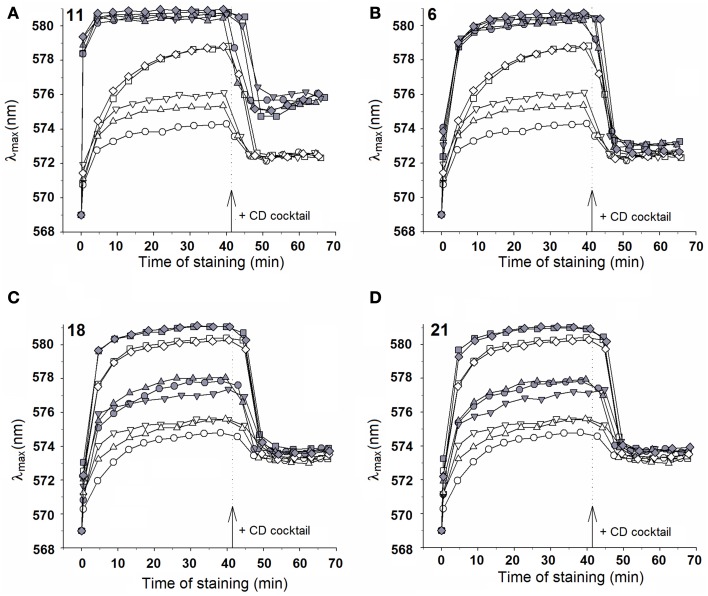
**Effect of selected compounds on the membrane potential and activity of MDR pumps of *S. cerevisiae***. Staining curves of AD1-3 (squares), AD23 (diamonds), AD12 (circles), AD13 (inverted triangles), and US50-18C (triangles) cells. Empty symbols–no compound added; full symbols–compounds added 10 min before diS-C_3_(3) at following concentrations: 1 μM **(A)**, 10 μM **(B–D)**. Dotted lines with arrows indicate the addition of the CD cocktail.

Selected chalcones **6**, **11**, **18**, and **21** were tested in a broad range of concentrations, from 0.1 μM to 20 μM. The representative staining curves obtained for chalcone derivatives are presented in Figure 1. Cells treated with **11** (Figure 1A) demonstrated the highest initial rate of staining, indicating rapidly increasing cell surface permeability for the probe. It should be noted that the magnitude of the red shift induced by **11** action on AD1-3 cells (drug efflux pump-free) was concentration-dependent and was observed even at concentration as low as 0.1 μM (graphs not shown). Addition of the CD cocktail caused lower drop of λ_max_, indicating partial cell damage.

Three compounds, **6**, **18**, and **21**, caused hyperpolarization of the cell membrane but did not damage the cells. Increased staining of pump-expressing cells after their exposure to compounds in comparison to chalcone-free controls was caused by both hyperpolarization and inhibition of the probe export. A strong inhibition of diS-C_3_(3) efflux by **6** and **11** was observed in the case of cells expressing Pdr5p or Snq2 (Figures 1A,B), presence of chalcones **18** and **21** led only to a partial inhibition of the probe export (Figures 1C,D).

### Effect of chalcone derivatives on Nile Red accumulation

**Figure 2:**
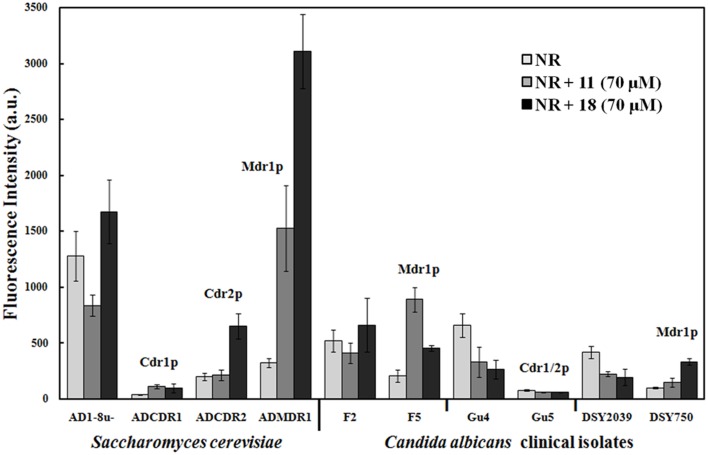
**Influence of selected chalcones on Nile Red accumulation in drug efflux pump-free and MDR yeast cells**. Cells were incubated for 30 min with Nile Red and chalcones and then fluorescence was measured with a flow cytometer. Values are the means of three independent experiments. Bars represent SD.

Nile Red (NR) is a fluorogenic substrate of *C. albicans* ABC transporters Cdr1p and Cdr2p and the MFS transporter Mdr1p (Ivnitski-Steele et al., 2009). The probe was used in a flow cytometry-based assay to measure influence of chalcone derivatives on NR accumulation in yeast cells. Biological models used in these studies were: the *Saccharomyces cerevisiae* AD1-8u^−^ strain and its fluconazole-resistant transformants: ADCDR1, ADCDR2, and ADMDR1, along with the matched pairs of clinical *Candida* isolates, F2/F5, Gu4/Gu5, and DSY2039/DSY750. Cells were loaded with NR and levels of fluorescence derived from NR accumulated by chalcone treated pump-expressing cells was compared to that of the pump-deficient cells. As shown in Figure 2, significantly lower level of NR-derived fluorescence was measured in all resistant cells, comparing to their pump-deficient counterparts, what indicates an active efflux of NR from the former. The ADMDR1 cells accumulated approximately tenfold more, the ADCDR2 cells threefold more and ADCDR1 twofold more of NR in the presence of compound **18** at 70 μM (~ 28 μg/mL) than the AD1-8u^−^ cells. Significant accumulation NR in ADMDR1 was also induced by **21**. Accumulation of NR in ADCDR1 and ADCDR2 cells remained unaffected by **11**. Further studies showed that **21** and **18** at concentrations as low as 0.5 μg/mL still strongly inhibited NR efflux from ADMDR1, causing a twofold higher accumulation of the probe in comparison AD1-8u^−^ (data not shown). Compound **6** at 70 μM caused significant accumulation of NR exclusively in ADCDR2 and ADCDR1 (2.5 × and 2 ×, respectively in comparison to AD1-8u^−^), with no effect on ADMDR1 (details not shown).

The inhibitory effect of **21** and **18** on MDR1p-mediated efflux was confirmed in the model of clinical *Candida* isolates. Compound **21** inhibited NR efflux only from the cells of the F5 and DSY750 strains overexpressing the *MDR1* gene, where respectively fivefold and twofold increase in NR-derived fluorescence was observed. Surprisingly enough, compound **18** was also found to interfere only with Mdr1p-mediated efflux. In F5 cells, accumulation of NR increased two times and in DSY750, three times. Both compounds were not able to inhibit NR efflux from Gu5 isolate overproducing Cdr1p and Cdr2p proteins. Some accumulation of NR in Gu5 but not in F5 and DS750 was observed in the presence of **6** (details not shown). Interestingly, in all sensitive strains, presence of **21** resulted in lower NR accumulation than presence of **18**.

### Effect of chalcones on rhodamine 6G efflux

In order to get more data characterizing chalcone derivatives as substrates of membrane multidrug transporters, their effect on efflux from yeast cells of another probe, Rhodamine 6G (R6G), which is a known substrate of Cdr1p/Cdr2p but not of the Mdr1p transporter, was investigated. *Saccharomyces cerevisiae* ADCDR1, ADCDR2 and control AD1-8u^−^ cells were first de-energized in presence of 2-deoxy-D-glucose and 2,4-dinitrophenol, then loaded with R6G (final concentration 10 μM) along with a compound tested and subsequently activity of drug-effluxing ABC-type proteins was triggered by glucose addition. Concentration of the effluxed R6G was determined after 30 min in supernatants obtained after cell harvesting.

The ADCDR1 and ADCDR2 cells extruded five times more R6G than the AD1-8u^−^ cells, thus confirming that this compound is indeed a substrate of the Cdr1p and Cdr2p drug transporters. Presence of compounds **6**, **18**, and **21** at 100 μM (~ 40 μg/mL) did not change the amount of R6G released from AD1-8u^−^ cells. Addition of **21** at 100 μM to the suspension of ADCDR1 or ADCDR2 cells inhibited R6G efflux only in about 5 ± 9% and 8 ± 4%, respectively, whereas presence of **6** and **18** at the same concentration resulted in 45 ± 10% and 38 ± 9% inhibition of the probe efflux from ADCDR2 and in 27 ± 8% and 33 ± 6% inhibition of R6G export from ADCDR1, compared to the untreated cells. These results corresponded well with those from the Nile Red assay and showed that compounds **6** and **18** may block to some extent the Cdr1p/Cdr2p-mediated efflux from recombinant *S. cerevisiae*, while presence of **21** had almost no effect on the activity of this drug transporter.

### Influence of chalcones on cell wall structure

**Figure 3:**
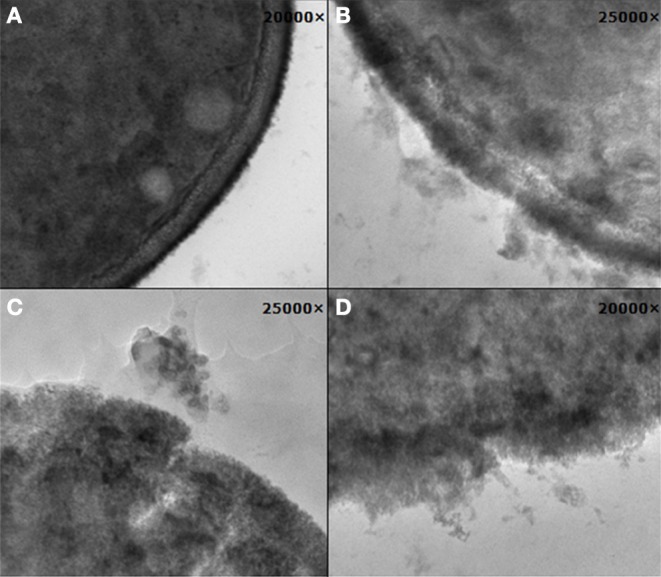
**Changes in the cell surface of *C. albicans* cells observed by transmission electron microscopy: (A) control cells; (B–D) cells treated with 11, 10 μg/mL, for 3, 6, and 9 h**.

The effect of selected chalcones on morphology and ultrastructure of *C. albicans* cells was investigated using transmission electron microscopy (TEM). The morphological alterations observed in cells treated with **6, 11, 18**, or **21** at 10 μg/mL were documented by microphotographs and some of these photos are shown in Figure 3. The cross-section of untreated cells reveals a typical morphology with an intact cell wall and cytoplasmic membrane, separated by a low-density space (Figure 3A). Treatment of cells with compounds **6, 18**, and **21** did not cause any visible changes, as the cross-sections of chalcone-treated cells looked very similar to those of the untreated control (photos not shown). This is not surprising, since 10 μg/mL is well below the MIC value of these compounds (64 μg/mL). On the other hand, **11** induced significant morphological changes, which ranged from some discrete alterations to the total destruction of the outer layers of fungal cells (Figures 3B–D). A common alteration observed after treatment with compound **11** was a loss of a typical layered structure and discontinuity or even disappearance of the cytoplasmic membrane (Figures 3C,D). Other changes comprised appearance of the irregular cell surfaces, loss of cell-wall integrity and penetrating lesions of the wall with an apparent shedding of the cell components (Figure 3C).

## Discussion

**Figure 4:**
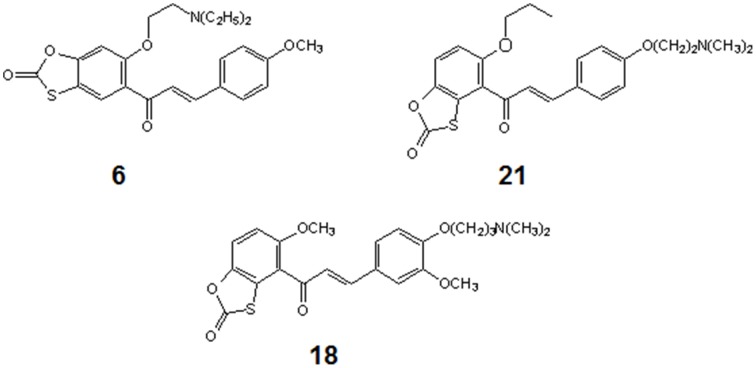
**Structures of chemosensitizers of MDR yeasts selected in this study**.

Three out of 27 chalcones studied in this work (Figure 4) appeared effective chemosensitizers, able to restore to large extent sensitivity to fluconazole of MDR *C. albicans* strains. Compound **21** effectively chemosensitized cells overexpressing the MFS-type Mdr1p, compound **6** did the same with cells FLC-resistant due to the activity of ABC-type drug transporters and derivative **18** partially reversed fluconazole resistance mediated by both types of yeast drug efflux pumps. This is worth mentioning that compounds **6**, **18**, and **21** demonstrated low *in vitro* mammalian toxicity against different cell lines in the tissue cultures (Konieczny et al., 2007a,b,c), what makes them promising candidates for clinical application as agents augmenting antifungal chemotherapy with FLC of infections caused by MDR *C. albicans*. On the other hand, the chemosensitizing potential of **11** seems questionable, since this compound exhibits a strong growth inhibitory and fungicidal effect at relatively low concentrations. In our previous studies we provided evidence for inhibition of chitin biosynthesis as a molecular basis of fungistatic effect of **11** and for inhibition of β(1 → 3)glucan synthase resulting in fungicidal action of this chalcone derivative (Ła̧cka et al., 2011). The latter has been now confirmed by the loss of continuity of *C. albicans* cells and the appearance of the cell wall defects, followed by leakage of cell components, demonstrated by TEM upon the action of **11** at concentration well above its MIC and close to the MFC value. Destruction of *S. cerevisiae* cells treated with **11**, revealed by the results of experiments involving the diS-C_3_(3) fluorescent probe, provides another evidence confirming this hypothesis. Inhibition of chitin biosynthesis by **11** at concentrations close to its MIC (Ła̧cka et al., 2011) seems to constitute a molecular basis for the observed synergism of **11** and FLC, similarly as it was shown previously for combination of the known inhibitor of chitin synthase nikkomycin and azole antifungals (Milewski et al., 1991).

It is not clear why the chemosensitizing potency of **6**, **18**, and **21** is much better than that of their other close structural analogs tested by us. The only characteristic common structural pattern observed here is presence of the 4′-dimethylaminoalkoxy substituent in ring B (compounds **11**, **16**, **18**, and **21**) that seems beneficial for the chemosensitizing efficacy (**18** and **21**) or high antifungal activity (**11**) of type 2 oxathiolone fused chalcones but this effect is abolished when the similar substituent is also present in the A ring (**16**).

Results of experiments employing Rhodamine 6G and Nile Red showed that some chalcones studied effectively interfered with extrusion of the fluorescent probes by the ABC and/or MFS proteins. Compounds **18** and **6** inhibited the efflux of Nile Red by Cdr1p, Cdr2p, and Mdr1p, export of Rhodamine 6G by the Cdr1p and Cdr2p transporters and efflux of diS-C_3_(3) from the *S. cerevisiae* strain overexpressing *PDR5*. On the other hand, **21** did not affect the Rhodamine 6G and Nile Red efflux mediated by Cdr1p/Cdr2p efflux and poorly affected export of diS-C_3_(3) from *S. cerevisiae* strains overexpressing *PDR5* and/or *SNQ2*, while it effectively inhibited the efflux of Nile Red from strains overexpressing *MDR1*. An inhibitory effect of **6** on Pdr5p- and Snq2p-mediated efflux of the diS-C_3_(3) probe and a very slight inhibition of the Cdr1p/Cdr2p-derived ATPase activity under *in vitro* conditions indicates its possible inhibitory activity against different ABC-type yeast drug transporters, probably not resulting from interaction with the ATP-binding domains. Previously it was shown that 4-alkoxychalcones (structure different from that of compounds described in this study) bind to the ATP binding site and to the steroid binding site of mammalian ABC-type drug transporter P-glycoprote (Conseil et al., 1998). It is possible therefore that compound **6** may also bind to more than one site in the ABC-type yeast drug transporters.

The fact that some chalcones effectively prevented extrusion of particular fluorescent probes from MDR *C. albicans* cells and chemosensitized MDR cells to FLC but on the other hand, their intrinsic anticandidal activity against FLC-resistant MDR cells, was very similar or the same as against FLC-sensitive cells, may indicate that these compounds bind to the MDR proteins outside their substrate-binding sites and prevent binding of probes or fluconazole to these sites but are not effectively extruded by the drug efflux pumps. In summary, the observed effect of sensitization of resistant strains of *Candida albicans* to FLC in the presence of chalconic chemosensitizers, most likely results from a non-competitive inhibition of drug efflux proteins, especially those of the MFS-type, although this hypothesis should be further verified.

### Conflict of interest statement

The authors declare that the research was conducted in the absence of any commercial or financial relationships that could be construed as a potential conflict of interest.
